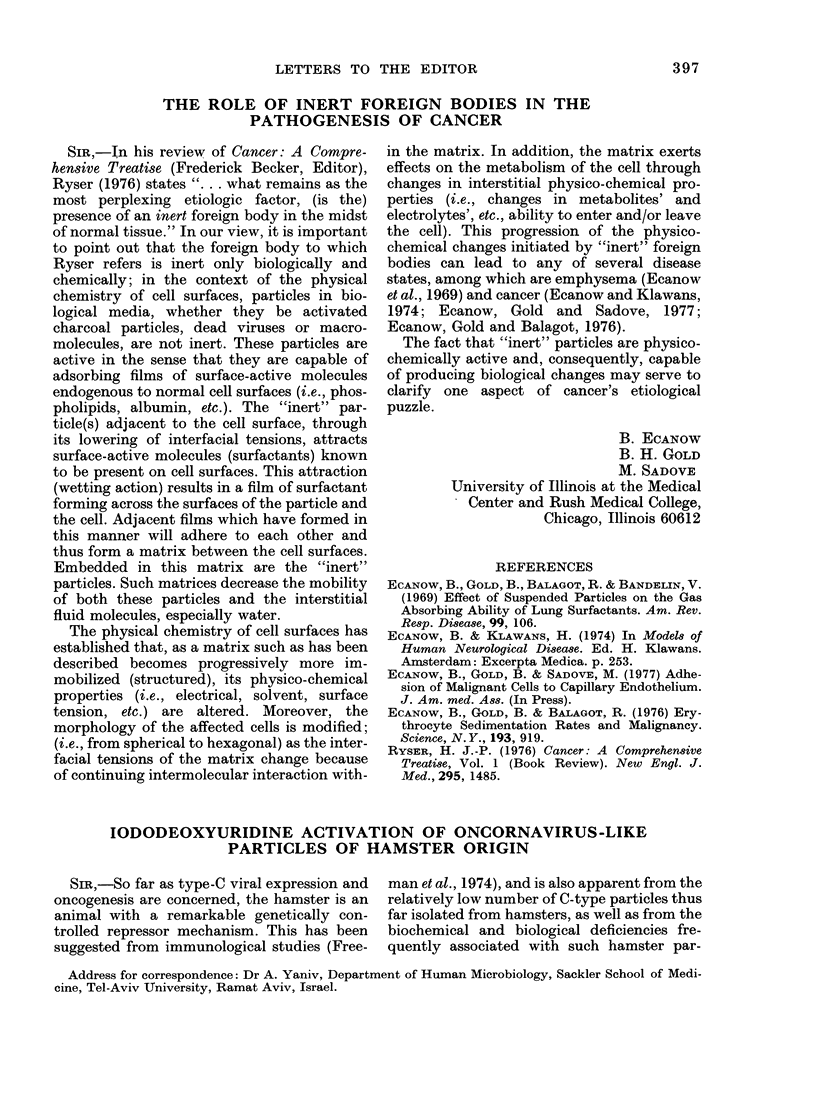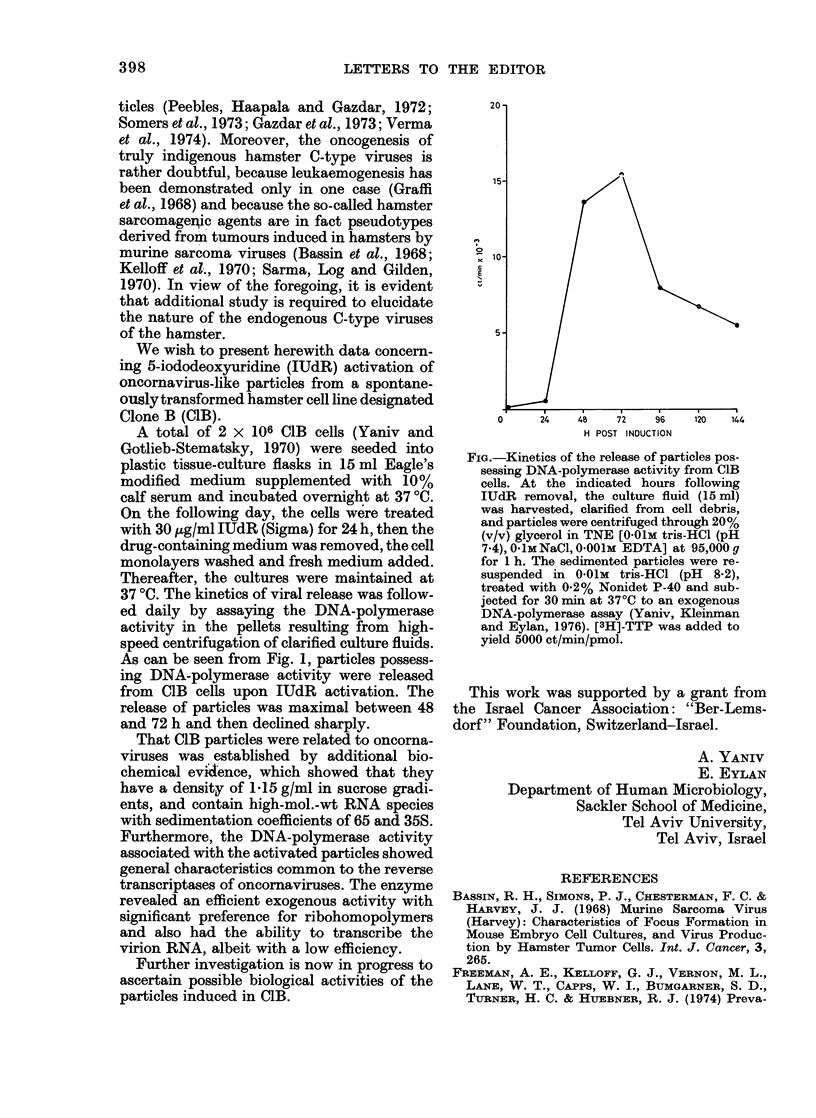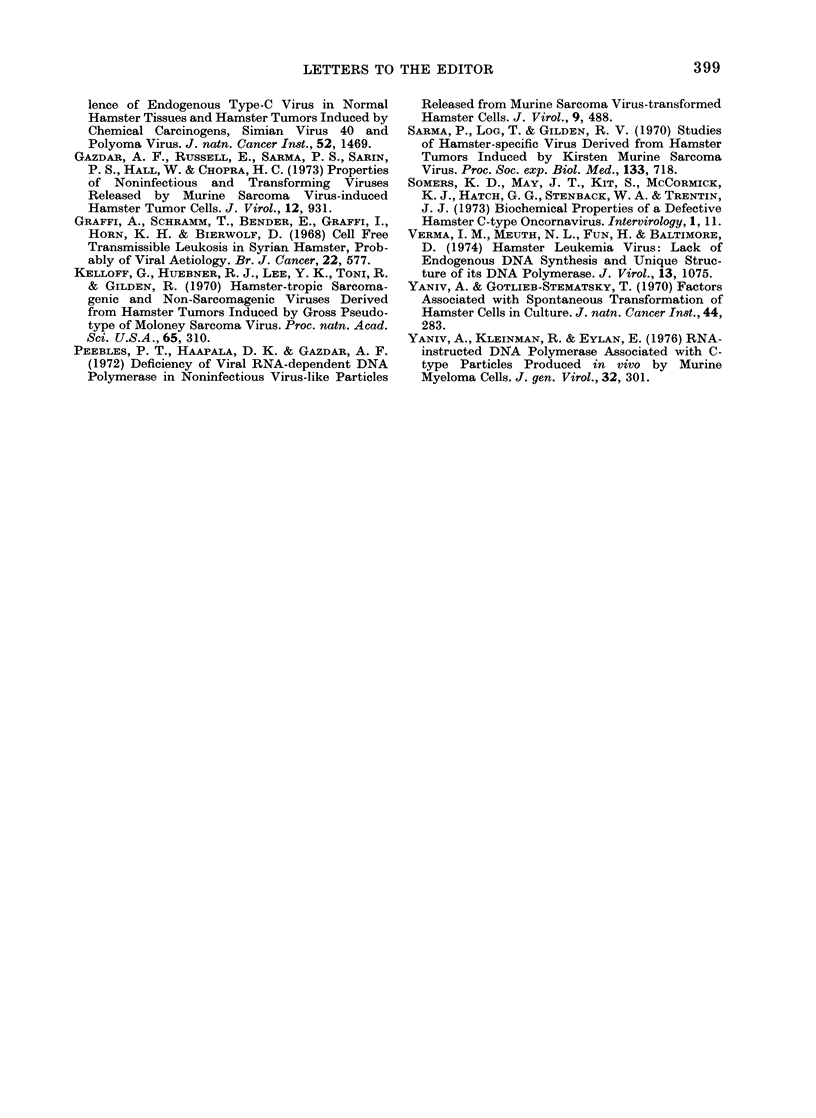# Iododeoxyuridine activation of oncornavirus-like particles of hamster origin.

**DOI:** 10.1038/bjc.1977.208

**Published:** 1977-09

**Authors:** A. Yaniv, E. Eylan


### IODODEOXYURIDINE ACTIVATION OF ONCORNAVIRUS-LIKE PARTICLES OF HAMSTER ORIGIN

SIR,—So far as type-C viral expression and oncogenesis are concerned, the hamster is an animal with a remarkable genetically controlled repressor mechanism. This has been suggested from immunological studies (Freeman *et al.*, 1974), and is also apparent from the relatively low number of C-type particles thus far isolated from hamsters, as well as from the biochemical and biological deficiencies frequently associated with such hamster par-

Address for correspondence: Dr A. Yaniv, Department of Human Microbiology, Sackler School of Medicine, Tel-Aviv University, Ramat Aviv, Israel.

ticles (Peebles, Haapala and Gazdar, 1972; Somers *et al.*, 1973; Gazdar *et al.*, 1973; Verma *et al.*, 1974). Moreover, the oncogenesis of truly indigenous hamster C-type viruses is rather doubtful, because leukaemogenesis has been demonstrated only in one case (Graffi *et al.*, 1968) and because the so-called hamster sarcomagenic agents are in fact pseudotypes derived from tumours induced in hamsters by murine sarcoma viruses (Bassin *et al.*, 1968; Kelloff *et al.*, 1970; Sarma, Log and Gilden, 1970). In view of the foregoing, it is evident that additional study is required to elucidate the nature of the endogenous C-type viruses of the hamster.

We wish to present herewith data concerning 5-iododeoxyuridine (IUdR) activation of oncornavirus-like particles from a spontaneously transformed hamster cell line designated Clone B (ClB).

A total of $2 \times 10^6$ ClB cells (Yaniv and Gotlieb-Stematsky, 1970) were seeded into plastic tissue-culture flasks in 15 ml Eagle's modified medium supplemented with 10% calf serum and incubated overnight at 37 °C. On the following day, the cells were treated with 30 $\mu$g/ml IUdR (Sigma) for 24 h, then the drug-containing medium was removed, the cell monolayers washed and fresh medium added. Thereafter, the cultures were maintained at 37 °C. The kinetics of viral release was followed daily by assaying the DNA-polymerase activity in the pellets resulting from high-speed centrifugation of clarified culture fluids. As can be seen from Fig. 1, particles possessing DNA-polymerase activity were released from ClB cells upon IUdR activation. The release of particles was maximal between 48 and 72 h and then declined sharply.

That ClB particles were related to oncornaviruses was established by additional biochemical evidence, which showed that they have a density of 1·15 g/ml in sucrose gradients, and contain high-mol.-wt RNA species with sedimentation coefficients of 65 and 35S. Furthermore, the DNA-polymerase activity associated with the activated particles showed general characteristics common to the reverse transcriptases of oncornaviruses. The enzyme revealed an efficient exogenous activity with significant preference for ribohomopolymers and also had the ability to transcribe the virion RNA, albeit with a low efficiency.

Further investigation is now in progress to ascertain possible biological activities of the particles induced in ClB.

FIG.—Kinetics of the release of particles possessing DNA-polymerase activity from ClB cells. At the indicated hours following IUdR removal, the culture fluid (15 ml) was harvested, clarified from cell debris, and particles were centrifuged through 20% (v/v) glycerol in TNE [0·01M tris-HCl (pH 7·4), 0·1M NaCl, 0·001M EDTA] at 95,000 *g* for 1 h. The sedimented particles were resuspended in 0·01M tris-HCl (pH 8·2), treated with 0·2% Nonidet P-40 and subjected for 30 min at 37°C to an exogenous DNA-polymerase assay (Yaniv, Kleinman and Eylan, 1976). [$^3$H]-TTP was added to yield 5000 ct/min/pmol.

This work was supported by a grant from the Israel Cancer Association: "Ber-Lemsdorf" Foundation, Switzerland–Israel.

A. YANIV
E. EYLAN
Department of Human Microbiology,
Sackler School of Medicine,
Tel Aviv University,
Tel Aviv, Israel

REFERENCES

BASSIN, R. H., SIMONS, P. J., CHESTERMAN, F. C. & HARVEY, J. J. (1968) Murine Sarcoma Virus (Harvey): Characteristics of Focus Formation in Mouse Embryo Cell Cultures, and Virus Production by Hamster Tumor Cells. *Int. J. Cancer*, **3**, 265.

FREEMAN, A. E., KELLOFF, G. J., VERNON, M. L., LANE, W. T., CAPPS, W. I., BUMGARNER, S. D., TURNER, H. C. & HUEBNER, R. J. (1974) Prevalence of Endogenous Type-C Virus in Normal Hamster Tissues and Hamster Tumors Induced by Chemical Carcinogens, Simian Virus 40 and Polyoma Virus. *J. natn. Cancer Inst.*, **52**, 1469.

GAZDAR, A. F., RUSSELL, E., SARMA, P. S., SARIN, P. S., HALL, W. & CHOPRA, H. C. (1973) Properties of Noninfectious and Transforming Viruses Released by Murine Sarcoma Virus-induced Hamster Tumor Cells. *J. Virol.*, **12**, 931.

GRAFFI, A., SCHRAMM, T., BENDER, E., GRAFFI, I., HORN, K. H. & BIERWOLF, D. (1968) Cell Free Transmissible Leukosis in Syrian Hamster, Probably of Viral Aetiology. *Br. J. Cancer*, **22**, 577.

KELLOFF, G., HUEBNER, R. J., LEE, Y. K., TONI, R. & GILDEN, R. (1970) Hamster-tropic Sarcomagenic and Non-Sarcomagenic Viruses Derived from Hamster Tumors Induced by Gross Pseudotype of Moloney Sarcoma Virus. *Proc. natn. Acad. Sci. U.S.A.*, **65**, 310.

PEEBLES, P. T., HAAPALA, D. K. & GAZDAR, A. F. (1972) Deficiency of Viral RNA-dependent DNA Polymerase in Noninfectious Virus-like Particles Released from Murine Sarcoma Virus-transformed Hamster Cells. *J. Virol.*, **9**, 488.

SARMA, P., LOG, T. & GILDEN, R. V. (1970) Studies of Hamster-specific Virus Derived from Hamster Tumors Induced by Kirsten Murine Sarcoma Virus. *Proc. Soc. exp. Biol. Med.*, **133**, 718.

SOMERS, K. D., MAY, J. T., KIT, S., MCCORMICK, K. J., HATCH, G. G., STENBACK, W. A. & TRENTIN, J. J. (1973) Biochemical Properties of a Defective Hamster C-type Oncornavirus. *Intervirology*, **1**, 11.

VERMA, I. M., MEUTH, N. L., FUN, H. & BALTIMORE, D. (1974) Hamster Leukemia Virus: Lack of Endogenous DNA Synthesis and Unique Structure of its DNA Polymerase. *J. Virol.*, **13**, 1075.

YANIV, A. & GOTLIEB-STEMATSKY, T. (1970) Factors Associated with Spontaneous Transformation of Hamster Cells in Culture. *J. natn. Cancer Inst.*, **44**, 283.

YANIV, A., KLEINMAN, R. & EYLAN, E. (1976) RNA-instructed DNA Polymerase Associated with C-type Particles Produced *in vivo* by Murine Myeloma Cells. *J. gen. Virol.*, **32**, 301.